# Secondary membranous nephropathy associated to autoimmune polyglandular syndrome Type II

**DOI:** 10.1080/0886022X.2023.2237110

**Published:** 2023-07-21

**Authors:** Alexa Golbus, Krunal Patel, Natalie Freidin

**Affiliations:** Department of Medicine, Medical University of South Carolina, Charleston, SC, USA

Images:


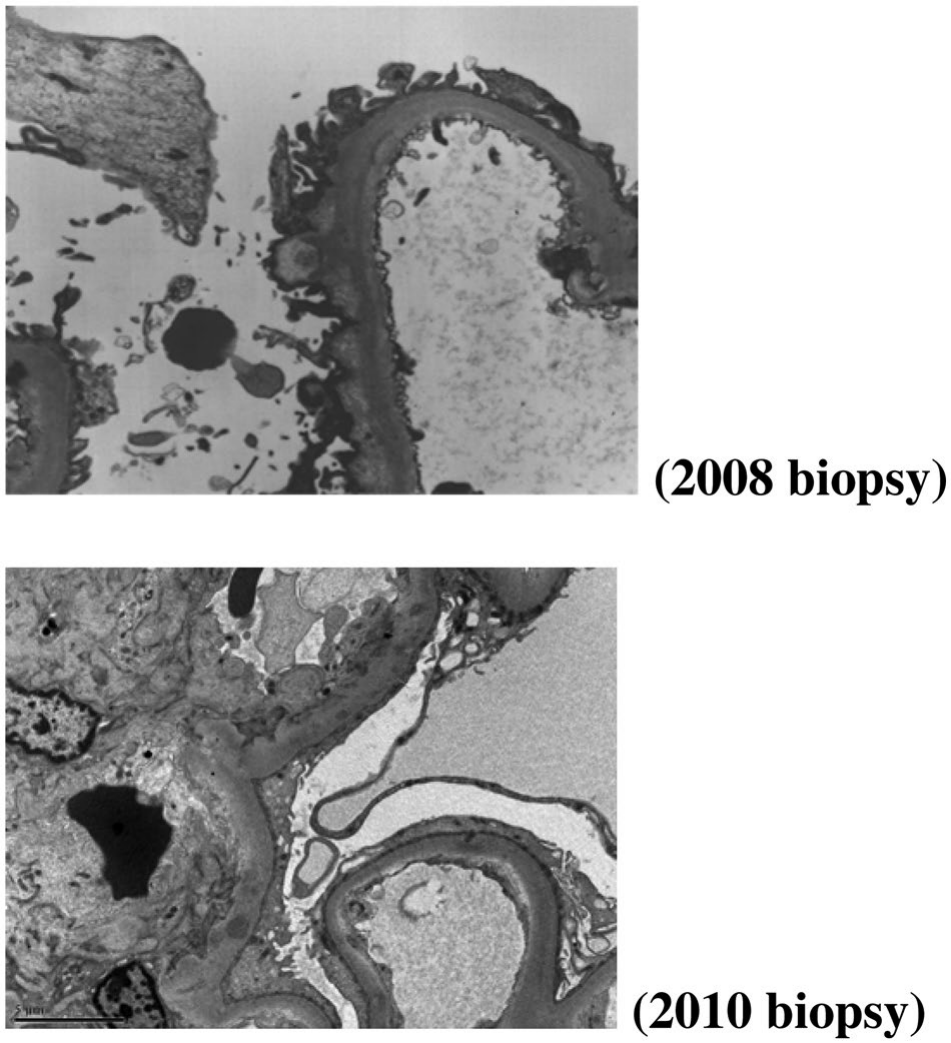


Autoimmune polyglandular syndromes (APS) are characterized by the presence of two or more coexisting autoimmune mediated endocrinopathies, including Addison’s disease and autoimmune thyroid disease or Type 1 diabetes mellitus. There are limited reports of autoimmune thyroid disease resulting in a secondary membranous nephropathy. While Hashimoto’s thyroiditis is reported to cause glomerular involvement in 10-30% of affected patients, and a secondary membranous nephropathy in 20% of such patients, there are very limited reports of Graves’ disease causing secondary membranous nephropathy and evidence that renal deposition of thyroglobulin or thyroperoxidase may be involved in these cases [1,2].

Known antigens associated with primary membranous nephropathy include PLA2R and THSD7A, which account for 60% of all cases, as well as newly identified antigens including exotosin 1 and 2 (EXT 1, 2), neural EGF-1 like protein (NELL-1), semaphorin 3B (Sema3B), and protocadherin 7 (PCDH7) [3]. PLA2R, THSD7A, and NELL-1 are causative for primary membranous nephropathy, while EXT1 and 2 are associated with autoimmune disease and secondary membranous nephropathy [4].

Graves’ disease, which is characterized by thyrotoxicosis, is an autoimmune thyroid disease in which lymphocytic infiltration of the thyroid gland and production of antibodies to the thyrotropin receptor (TSHr antibodies), results in overproduction of thyroid hormone. Graves’ disease, when cooccurring with additional autoimmune endocrinopathies, may be part of an APS. In 40-50% of patients with adult-onset APS, additional non glandular autoimmune diseases are seen including atrophic gastritis, pernicious anemia, atopic eczema, alopecia areata, myasthenia gravis, systemic lupus erythematosus, rheumatoid arthritis, and autoimmune hepatitis. Primary hypogonadism, hypoparathyroidism, or hypopituitarism may also be seen [5].

While there are limited reports of autoimmune thyroid disease resulting in a secondary membranous nephropathy, to our knowledge this is the first report of secondary membranous nephropathy in a patient with APS Type II.

A 31-year-old male with history of Grave’s disease status-post radioactive iodine therapy, Wolff-Parkinson-White syndrome, and gouty arthritis presented to his primary care physician for annual follow-up in 2008 and routine labs showed an elevated Creatinine of 1.8 mg/dL. Further workup showed 3+ proteinuria on urinalysis and 3.57 g of protein on 24-h urine protein collection. Renal biopsy performed for nephrotic-range proteinuria revealed acute tubular injury, glomerulomegaly with perihilar Focal Segmental Glomerulosclerosis (FSGS), and immunofluorescence and ultrastructural evidence of resolving membranous nephropathy. Biopsy was PLA2R and THSD7A negative. ANA, hepatitis panel, and complements studies were all negative. Patient had persistence of proteinuria and progression to CKD stage III, but no further etiologies or treatments were pursued.

Approximately 6 months later, our patient was admitted for hyperkalemia (7.9) and hyponatremia (130) found on outpatient labs. Cosyntropin test with no response and elevated adrenal antibodies led to diagnosis of Addison’s disease. He was started on hydrocortisone and fludrocortisone acetate for hormone replacement. Given combination of Graves’ disease and Addison’s disease, patient was diagnosed with APS Type II. One year after initial renal biopsy, he had persistent proteinuria (2.5 g/day) and repeat biopsy revealed glomerulomegaly with FSGS and ultrastructural evidence of resolving membranous nephropathy. Again, no treatment pursued given findings.

Seven years following his second renal biopsy, at the age of 40, our patient presented to the ED for further evaluation following two weeks of worsening fatigue, weakness, metallic taste, and muscle cramping. Laboratory evaluation revealed a creatinine of 23 mg/dL and BUN 165 mg/dL. No demonstration of corrections or reversibility of his kidney function and thus he was declared ESRD and started on hemodialysis. In this same year, our patient was also diagnosed with primary hypogonadism.

Following ESRD declaration, patient completed transplant evaluation and underwent living unrelated kidney transplant on 7/12/2018. There were no intra or post-operative complications and creatinine at discharge was 1.79. One week following transplant, patient was admitted to the hospital for AKI with creatinine 3.7. Repeat renal biopsy revealed severe T-cell mediated rejection with extensive interstitial inflammation, severe tubulitis and superficial endothelitis that was consistent with TCMR grade 2 A/Banff 2 A rejection. Patient completed course of methylprednisolone and thymoglobulin for rejection management with improvement in creatinine. Currently, patient with CKD4 and baseline creatinine 1.5-1.7 and on tacrolimus for post transplant immunosuppression.

APS disorders are caused by T cell dependent and B cell mediated autoimmunity resulting in anti-receptor mediated autoimmune disorders. Loss of self-tolerance due to cellular and organ specific autoantibody mediated autoimmune responses ultimately lead to the development of the various endocrinopathies seen in APS. Dysregulation of CD4+, CD25+, FoxP3+ T regs result in various autoimmune disorders. Without the suppression of Tregs, auto antigens target the TSH receptor in thyrocytes leading to Graves’ disease and auto antigens to 21 hydroxylase and CYP450scc target the adrenal cortex leading to Addison’s disease [5].

While infections, drugs, tumors, and autoimmune diseases such as SLE are known to cause secondary membranous nephropathy, there are limited reports of Graves’ disease causing secondary membranous nephropathy. The mechanism by which this occurs is not fully known, but is thought to involve thyroglobulin causing an antigen-antibody reaction resulting in complement activation with immune complexes then depositing in the glomeruli, and/or thyroglobulin directly binding to the glomeruli and forming immune complexes [2]. While membranous nephropathy secondary to Graves’ disease is a rarely reported phenomenon, in a few prior documented cases renal biopsy stained positively for thyroglobulin and TPO, indicating that deposition of such in the kidney was responsible for the secondary membranous nephropathy [2,6]. Alternatively, the antigens EXT 1 or 2 may be involved in our case, as these antigens are associated with autoimmune induced secondary membranous nephropathy [4]. However, our patient’s renal biopsies were not stained for either thyroid antigens or EXT 1 or 2.

Our patient’s development of several autoimmune disorders and endocrinopathies including Graves’ disease, Addison’s disease, and hypogonadism, along with membranous nephropathy, supports an underlying autoimmune etiology. However, it remains unclear whether Graves’ disease in the setting of APS caused a secondary membranous nephropathy, or whether an antigen antibody process is directly responsible for both our patient’s APS and membranous nephropathy.
